# Resource-efficient photonic networks for next-generation AI computing

**DOI:** 10.1038/s41377-024-01717-6

**Published:** 2025-01-04

**Authors:** Ilker Oguz, Mustafa Yildirim, Jih-Liang Hsieh, Niyazi Ulas Dinc, Christophe Moser, Demetri Psaltis

**Affiliations:** EPFL, Institute of Electrical and Micro Engineering, Lausanne, Switzerland

**Keywords:** Optoelectronic devices and components, Nonlinear optics

## Abstract

Current trends in artificial intelligence toward larger models demand a rethinking of both hardware and algorithms. Photonics-based systems offer high-speed, energy-efficient computing units, provided algorithms are designed to exploit photonics’ unique strengths. The recent implementation of cellular automata in photonics demonstrates how a few local interactions can achieve high throughput and precision.

Current artificial intelligence (AI) models based on neural networks are gaining previously inaccessible cognitive and creative abilities with the continuous increase in their scale. State-of-the-art models now tend to double their sizes every year, as shown in Fig. 1a, reaching trillions of parameters today. In addition to better performances in their training tasks, as the models are scaled up, they have also been observed to start performing new tasks that they were not trained for^1^. Fig. 1 illustrates this phenomenon, showing language models obtain capabilities outside of their training after reaching a certain level of complexity. This expanded skill set, coupled with wider adoption across various sectors, is driving a rapid increase in global computing resource and energy demands for AI, currently doubling every 100 days^2^. The corresponding environmental impact of this energy-hungry technology necessitates the development of more compact AI models and more efficient hardware, while maintaining high performance.

**Fig. 1:**
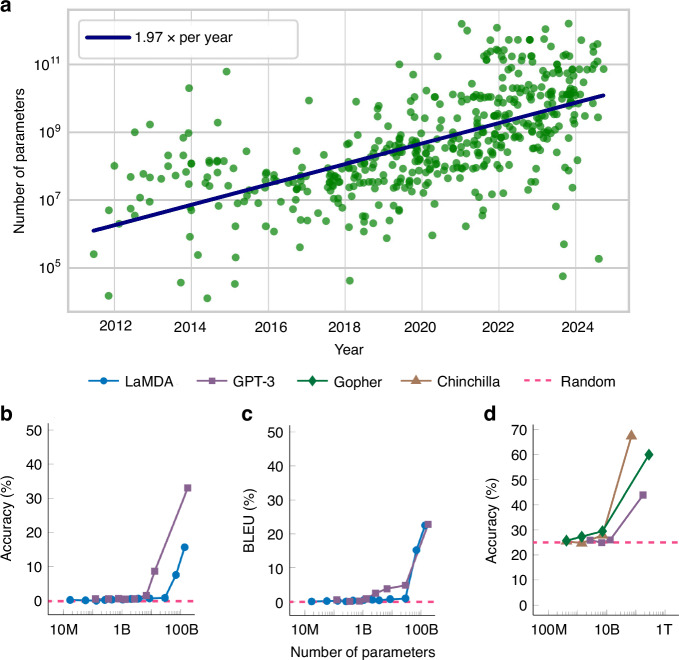
The trend and impact of the scale of artificial intelligence (AI) models. **a** The trend of the total number of parameters of the state-of-the-art AI models over time, each data point refers to such a model (Epoch (2024) – with major processing by Our World in Data). **b**–**d** Different examples of emergent capabilities in large-scale language models. As the scale of these models trained on generic language datasets increases, they become able to perform tasks beyond those for which they are explicitly trained. **b** Accuracy on arithmetic operations task^17^. **c** Translation accuracy between International Phonetic Alphabet and English^17^ **d** Accuracy on multitask language understanding, a benchmark containing 57 tasks, ranging from computer science to law^18^

Different machine learning methods address the goal of achieving competitive accuracies with smaller and lighter models. As one of the earlier techniques, pruning reduces the size of neural networks by determining less important connections after training and eliminating them^3^. Knowledge distillation trains a smaller model with the intermediate activations of a larger model, achieving similar performance with fewer parameters^4^. The method called quantization, which is simply decreasing the bit depth of model parameters and/or activations during inference, for instance from 16 bits to 8 bits, also resulted in larger throughput with the same computational resources^5^. Relying on randomly initialized, fixed hidden layers that do not require gradient-based training, Extreme Learning Machines (ELM)^6^ and reservoir computing^7^ decrease the number of trainable parameters. Another advantage of these architectures is the possibility of low-power, high-dimensional and parametric physical events to perform their fixed layers with high efficiency.

Alongside advances in AI algorithms, the use of alternative modalities for hardware holds the potential to reduce the environmental impact of this technology. Photonics is one of the promising candidates since it can sustain larger bandwidths and lower losses compared to digital electronics. Mature photonic technologies, such as integrated and spatial light modulators, enable the implementation of various AI models, including fully programmable architectures^8,9^ and configurations with fixed layers, whose functionality comes from physical interactions such as multimode lasing^10^, nonlinear frequency conversion^11^ or random scattering^12^. Besides power efficiency, another advantage of high-dimensional nonlinear physical events is their suitability for computing complex tasks with a minimal number of parameters^13^. This advantage has been demonstrated with spatiotemporal nonlinearities in multimode fibers, the selection from a large set of readily available connectivities achieved the accuracy of artificial neural networks with over two orders of magnitude more parameters than the optical implementation^14^.

Compared to global connections in layers such as fully connected and attention, processing information with local connections in an AI model results in more compact architectures, one very popular and influential example being convolutional layers. Neural cellular automata (NCA), inspired by traditional cellular automata in which each cell of the system evolves according to local rules that depend on neighboring cell states, use differentiable, continuous-valued functions to define these interactions^15^. This design allows NCA to perform complex tasks through simple update rules. The “neural” or differentiable nature of NCA enables the definition of a downstream task for the local interactions and subsequent training of interaction weights accordingly.

In the study by Li et. al. from the California Institute of Technology, the downstream task was defined as the classification of the overall pattern formed by pixels (or “cells”, in the context of cellular automata), and a photonic system has achieved the implementation of the NCA^16^. The computational model depending on the recurrent updates to the individual cell values according to the interaction rules was proved to be a convenient match with the capabilities of photonics. As shown in Fig. 2, the various computational functionalities required by the algorithm were realized by different optical components. During inference, the fixed interactions between cells were implemented with a variable optical attenuator, while second harmonic generation in the periodically poled lithium niobate acts as the nonlinear activation function. The updated cell values were then detected and returned to the optical domain through a high-speed electro-optic modulator.

**Fig. 2:**
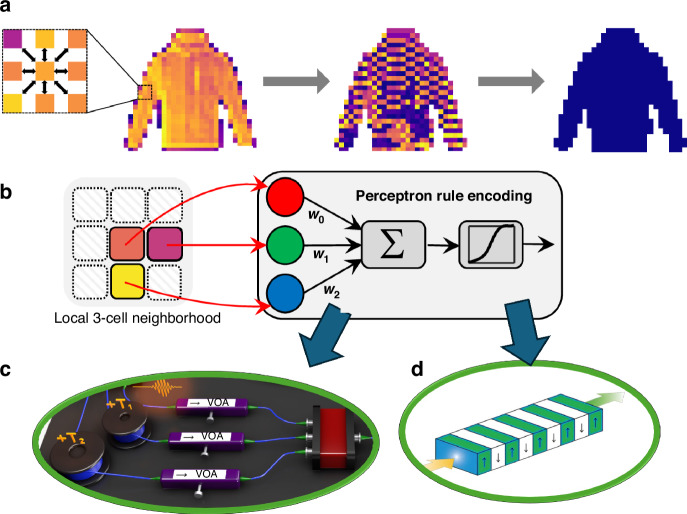
Working principle and experimental implementation of the Photonic Neural Cellular automata. **a** Working principle of neural cellular automata. Each pixel/cell interacts with its neighboring cells with a set of weights, trained with gradient descent. The final values of these cells represent an individual local decision about the global distribution. **b** The local interaction scheme behaves as a perceptron, whose output becomes the value of the cell in the next step. While the weighted sum is performed in photonics by the combination of the outputs of variable optical attenuators, **c** the pump depletion in a periodically poled lithium niobate waveguide, **d** serves as the nonlinear activation

Leveraging the immense data rate of the modulator, the optoelectronic system achieved predictions at a state-of-the-art rate of 1.3 μs per frame. This high throughput was further enabled by the simplicity of the local interaction model, that was defined by only 3 parameters, allowing each cell to compute its next state based on its current state and the states of its two neighbors. For the final binary classification, a majority “vote” was conducted across all cells, with classification as “1” if the majority of cells exceeded a threshold value and “0” otherwise. The classification precision reached 98.0%, closely matching the ideal simulation accuracy of 99.4%, due to the proposed mixture of experts approach’s resilience to experimental nonidealities, such as noise and device imperfections.

A remarkable finding of the paper by Li, et al., is that good accuracy can be obtained in the classification of images for the MNIST fashion database with 2 classes, In order to understand whether this is due to the specifics of the NCA architecture used, we implemented on the same database a more familiar multilayer network consisting of a single convolutional layer with a 2-by-2 kernel followed by a similar output classification layer. With a total of 7 parameters, this network achieved a similar 98.3% test accuracy while processing an image in 18.6 μs (instead of 1.3 μs) with a batch size of 1024, on an NVIDIA T4 GPU. We conclude, therefore, a strength of the photonic approach is that even compared to the highly optimized and parallelized GPU hardware, it was able to operate at a higher speed.

This photonic implementation of neural cellular automata (NCA) illustrates how photonics could address the explosion of model sizes and the environmental footprint of AI by utilizing high-speed hardware and physical interactions as computing units. Given the development of algorithms tailored to these platforms—considering the unique advantages and limitations of photonics rather than those of general-purpose digital hardware—photonics may offer a compelling solution. As demonstrated here, aligning the algorithm’s requirements with photonic capabilities enables implementations with high precision and throughput that could contribute to the scaling of AI sustainably.
